# Barriers and Enablers for Accessing Rehabilitation Services: Findings From the *Rehabilitation Choices* Study, Part 1—Healthcare Professionals' Perspectives

**DOI:** 10.1111/hex.14120

**Published:** 2024-06-21

**Authors:** Nicolette Hodyl, Gillian Mason, Karen Ribbons, Lucy Bailey, Adrian O'Malley, Tracy Ward, Stephen Ward, Michael Pollack, Michael Nilsson, Frederick Rohan Walker

**Affiliations:** ^1^ NSW Regional Health Partners Newcastle New South Wales Australia; ^2^ Centre for Rehab Innovations University of Newcastle Callaghan New South Wales Australia; ^3^ Hunter Medical Research Institute New Lambton Heights New South Wales Australia; ^4^ College of Health, Medicine and Wellbeing University of Newcastle Callaghan New South Wales Australia; ^5^ Hunter New England Area Health Service John Hunter Hospital, New Lambton Heights New South Wales Australia; ^6^ Lee Kong Chian School of Medicine Nanyang Technological University Nanyang Singapore

**Keywords:** barriers, decision‐making, enablers, healthcare professional, referral, rehabilitation

## Abstract

**Introduction:**

Globally, there is an increasing demand for quality medical rehabilitation services. This is the first article of a two‐part series showing the findings from the *Rehabilitation Choices* study in which the main aim was to understand the current landscape of decision‐making, enablers and barriers to access appropriate rehabilitation services in the Australian setting. In Part 1, these insights were sought from a healthcare professionals' perspective.

**Methods:**

This was an exploratory, qualitative study, using semi‐structured interviews with a discussion guide that was codesigned together with rehabilitation clinicians and rehabilitation researchers. Themes and sub‐themes were identified using an inductive approach.

**Results:**

We interviewed a heterogeneous group of 31 professionals who are involved in making referral decisions about rehabilitation or who design and deliver rehabilitation programs, including specialist rehabilitation physicians and other medical doctors across in‐patient, outpatient, and primary care settings, allied health professionals, rehabilitation service managers, nurses, multicultural health liaison officers and rehabilitation research scientists. Three key themes relevant to barriers and enablers to service access were identified from the data: defining rehabilitation; a lack of timely access to patient and rehabilitation service data; and patient diversity not expected by the system.

**Conclusions:**

Healthcare professionals who make decisions about rehabilitation referrals and services feel that it was necessary for them to keep up to date with information relating to rehabilitation services. There was some concern regarding what rehabilitation constituted and what services were available for different clinical indications. They also indicated that current systems did not consider diversity among patients' needs and goals. Their recommendations included the need for better communication pathways, improved referral systems and resources that could help provide best practice of rehabilitative care in the future.

**Patient or Public Contribution:**

Three study team members had a lived experience of rehabilitation as a patient or carer, and previous experience participating in qualitative research. They worked with the study team to codesign the recruitment strategy, participant‐facing communications, the interview discussion guide, and the approach to the conduct of activities with participants and in the interpretation and contextualization of findings and all were involved in writing this manuscript.

## 1 Introduction

Rehabilitation medicine is defined by the World Health Organisation (WHO) as the interventions designed to optimise functioning and reduce disability in individuals with health conditions in interaction with their environment [1, 2]. For simplicity, we will be referring to rehabilitation medicine as ‘rehabilitation’ in this study. A significant and growing number of people undergo rehabilitation each year, including following orthopaedic procedures and neurosurgery, following a stroke, cardiac infarction or cancer diagnosis [3, 4]. In 2019, an estimated 2.4 billion people worldwide had conditions that would benefit from different rehabilitation services, contributing to 310 million years of life lived with disability (YLD) [3]. The globally ageing population is contributing to the increasing burden of people with multiple chronic conditions, including cardiovascular disease and stroke, who will require rehabilitation services in the future [5, 6, 7]. In Australia, referral rates to extended inpatient rehabilitation services remain high [4], adding to the burden on healthcare services. In addition to the health impact, the socioeconomic benefits of rehabilitation are evident. Almost half of rehabilitation consumers, who receive either in‐hospital or external rehabilitation, report successful return to work [8]. In addition, a cost–benefit analysis of rehabilitation services showed a $AUD32.38 benefit for every $AUD1 spent on external services and an even greater claims saving of $AUD55.91 for in‐hospital rehabilitation support [8].

Despite its individual and economic societal benefits, rehabilitation has not been prioritised around the world and is often under‐resourced and not well‐organised [3]. Rehabilitation services require specialist medical and/or allied healthcare involvement for recovery. In Australia, although there is a large medical and allied health workforce engaged in the provision of rehabilitative services [9], the present system is not cohesive [10]. For several clinical indications, including poststroke [11], joint replacement [12] and chronic obstructive pulmonary disease (COPD) [13], it has been reported that there are no systematic or accepted processes for identifying who may benefit from rehabilitation, mapping of personalised rehabilitative needs or objectively monitoring the patient's individual rehabilitation progress. While there are guidelines, processes and policies to identify rehabilitation needs for stroke in Australia, there are no good systems for tracking implementation of or impact of this [14]. The fragmented array of public and private service providers caters to needs within a specific domain, for a specific period, with no mechanism or incentive to integrate with other service providers and often there is little feedback on individual patient progress across services along the recovery trajectory. In complex cases, as seen in stroke [15] and multiple sclerosis [16], it is necessary for multidisciplinary teams to be involved in rehabilitative care which can often be impacted by insufficient communication and referral pathways.

In relation to rehabilitation services, consumer choice is influenced by financial and social issues, awareness, understanding of rehabilitation options, the setting of rehabilitation, communication with healthcare professionals, consumer support and other factors [3, 17]. In the private sector, consumer choice has also been shown to be influenced by factors beyond the reported efficacy of rehabilitation types following arthroplasty, including clinical and social factors and a sense of entitlement [18]. A 2018 report by the Australian Royal College of Surgeons [19] identified that the evidence taken into consideration when making decisions around directing consumers to rehabilitation was poor. This concept supports an earlier 2012 report by the NSW Agency for Clinical Innovation (ACI), which called for the utilisation of more sophisticated and standardised measures to assess which consumers should receive rehabilitation [20]. Moreover, until the recent WHO definition, there was no clear consensus definition of rehabilitation, which adds to the confusion about what can be expected when seeking rehabilitation services. Consequently, over the last two decades, there has been a lack of understanding of rehabilitation needs and the specific services available and outcomes that can be achieved relevant to individual patient needs [21, 22]. Rehabilitation services need to be responsive to individuals' needs and concerns, and to do so, a better understanding of consumer decision‐making in accessing rehabilitation services is required. This was recognised in the Australasian Faculty of Rehabilitation Medicine (AFRM) Standards for the provision of Inpatient Adult Rehabilitation Medicine Services in Public and Private Hospitals and the ACI Principles to Support Rehabilitation Care [20, 23]. As indicated for inpatient rehabilitation services, the AFRM standards recommended that a continuous quality management strategy should include feedback sought from patients, their families and their carers, regarding service provision, and that there are clear post‐discharge planning strategies to support outpatient and community rehabilitation access [23].

Healthcare professionals have an important role in enabling access to rehabilitation services and seeking the best outcomes for their patients, and this is achieved through information‐sharing, promoting patients' self‐efficacy and providing relevant feedback during the rehabilitative process [24]. The knowledge of how health professionals make their decisions to refer people to hospitals, clinics or community‐based rehabilitation services is limited. A recent Australian study looking at rehabilitation for dementia patients found that healthcare professionals identified several challenges that influenced rehabilitation service delivery, including a lack of clarity of rehabilitation outcomes, healthcare professional role ambiguity and the lack of interdisciplinary teamwork [25]. Another Australian study explored clinician engagement with their rehabilitation patients and found that to develop patient‐centred goals, the interactions needed to encourage and allow patients to express their needs and preferences [26]. This presents challenges for healthcare professionals given their constraints in the current system, which is also mirrored in the global setting and highlights the demand to adopt a more patient‐centric model of care. A study that was the first to attempt to capture the views and decisions of UK healthcare providers outlines the growing body of European evidence to support alternative models of follow‐up rehabilitation for individual patients with cancer, largely involving joint nurse‐led/healthcare professional models of care that can offer a more holistic approach that is tailored to the specific needs of people with head and neck cancer [27]. Another study that explored healthcare provider views in the UK on rehabilitation service experiences after lung cancer surgery found that there was a benefit in providing personalised support and greater information to enhance patient outcomes [28].

### 1.1 Rationale for the *Rehabilitation Choices* Project

The *Rehabilitation Choices* study was conducted by The Centre for Rehab Innovations, New South Wales, Australia and funded by the Medibank Better Health Foundation, with additional support from the Priority Research Centre for Stroke and Brain Injury, University of Newcastle, New South Wales, Australia. This was an exploratory study, which sought to address the question of who currently influences consumer and healthcare professional decisions around engagement with medical rehabilitation services and how these processes can be streamlined for both consumers and healthcare professionals to improve the current rehabilitation model in Australia. We propose that an important step in understanding rehabilitation referrals and decisions in the Australian context lies in understanding factors that facilitate (enablers) or impede (barriers) access to different rehabilitation options. This information is important to support equitable access to effective and high‐quality rehabilitation pathways in both the private and public sectors. This requires an understanding of both the patient's and their healthcare team's needs, desires, perceptions and the environments in which they live and function. Armed with this information, we can identify current understanding and gaps in knowledge, that can be improved with targeted communications, cocreation of information and resources with patients and the development of patient and clinical guidelines to support the utilisation of the most appropriate and effective rehabilitation pathway. To address the aims of the current study, we adopted a qualitative research approach using semi‐structured interview questions to collect, analyse and interpret healthcare professionals' insights. A qualitative research methodology was adopted as it can provide a better understanding of complex problems that involve the social world and the beliefs and behaviours of the people within it. It can also allow for issues to be examined in detail, with a greater ability to uncover complexities, and can be more compelling than quantitative data, as it is based on personal experience [29].

## 2 Materials and Methods

The *Rehabilitation Choices* study included healthcare professionals, associated with different aspects of rehabilitative service delivery, to outline the barriers and enablers for rehabilitation decisions to support access to rehabilitative care and used the following methodology to collect, interpret and summarise the results.

### 2.1 Ethics Approval

Ethics approval was granted by the University of Newcastle Human Research Ethics Committee (approval H‐2020‐0324) on 4 November 2020.

### 2.2 Study Design

Two members of the research team were rehabilitation clinicians (M.N. and M.P.), and two members had lived experience in inpatient, outpatient and community‐based rehabilitation (N.H. and G.M.). Three additional research team members with lived experience of rehabilitation as a patient or carer and previous experience participating in the research were recruited to the team as dedicated consumer advisors for this project (A.O., S.W. and T.W.). Advisors were paid an honorarium commensurate with the Health Consumers NSW guidance. Three team members (G.M., S.W. and A.O.) had lived experience of disabilities, including physical and sensory disabilities, fatigue, vision impairment and communication disabilities. This allowed a collaborative multidisciplinary team approach in the design of all participant‐facing communications including the participant information sheet, study website and consent forms (included information in print and video formats to improve accessibility), the recruitment strategy and issues related to accessibility.

### 2.3 Participant Eligibility, Recruitment and Consent

Healthcare professionals were eligible to take part in the study if they refer patients to or provide rehabilitation services to consumers and had at least 5 years of experience working in their field of expertise. Participants were recruited through promotion on the social media accounts (Facebook, Twitter and LinkedIn) of our organisations [Centre for Rehab Innovations, Hunter Medical Research Institute (HMRI), University of Newcastle] and partner organisations, by direct email invitation to colleagues and relevant clinical networks and by word of mouth. Social media recruitment posts were listed (via the Facebook, Twitter and LinkedIn accounts of the Centre for Rehab Innovations, HMRI, University of Newcastle) and contact details for the research team were provided. Interested healthcare professionals were sent a participant information sheet and consent form and invited to discuss the study with the research team before giving consent to participate. Informed consent was obtained from all participants involved in the study before undertaking any study procedures. Participants were also invited to share any access requirements they needed. Videoconferencing and device test/set‐up or training sessions were provided by the research team and participants were able to choose to participate by telephone via videoconferencing at their discretion. Participants were sent a discussion guide to enable their preparation in advance.

### 2.4 Interview Guide

The guide was developed in consultation with a multidisciplinary research team which included rehabilitation researchers, clinicians and providers, who work across public and private services. The inclusion of two rehabilitation physicians (M.N. and M.P.) in the research team involved in the development of the interview guide, enabled the interviewers to use language appropriate for their research participants and enable questions relevant to the main aims of the study to be addressed. The guide was developed to facilitate interviewer‐guided discussions. In this study, we explored how healthcare professionals approached decisions in relation to rehabilitative services using qualitative semi‐structured interviews. Each interview was undertaken by one of two possible members of the research team (N.H. and G.M.), both having long‐standing experience in rehabilitation research and undertaking qualitative research methods and conducting interviews. The interviews took place between February and November 2021. Individual interviews (30 min) by videoconferencing or telephone were chosen as the best option to fit within the working schedule of healthcare professionals.

The interview guide raised the following questions with healthcare professionals:

1. Factors guiding decisions
  a. What factors about the patient do you consider when making referral decisions or designing rehab programs for people? (This question was tailored to the role of the participant)
  b. What other factors do you take into account when making these decisions?
2. Keeping abreast of information
  a. How do you keep abreast of information to help guide your decisions?
  b. From whom and where do you get information?
  c. Do you provide additional information or resources about rehab to your patients?
3. Barriers and Enablers
  a. What makes it difficult and what makes it easy for people to get access to the rehab they need?
  b. What do you think would help even further?
4. Additional information
  a. What else do you think I need to know?
  b. Is there an important question you think I've missed out on asking?

### 2.5 Data Analysis

To manage potential bias, the two interviewers (N.H., an academic researcher, and G.M., a clinician‐researcher) led the data analysis, and to establish positionality and reflect on potential researcher bias during data analysis, they met before interviews commenced to reflect on and document their own experiences and discuss the positionality of rehabilitation, as both professionals and as previous rehabilitation patients. This was used to reflect on the interpretation of the results in discussion with the wider investigative team including rehabilitation professionals (M.N. and M.P.), who contributed to the analysis and interpretation of the data generated. Key concepts were discussed and collated into patterns, and candidate categories and themes were developed using an inductive approach and a reflexive dialogue between the investigative team. Themes were further refined through an iterative process and, by consensus, merged into final themes. All participants were invited to attend an online forum where the preliminary results were shared, to make suggestions about themes and add to their contextualisation, before finalising the analysis.

## 3 Results

### 3.1 Participant Characteristics

Participants consisted of a multidisciplinary group of 31 healthcare professionals, involved in promoting or providing rehabilitation services to patients across primary care, public and private health settings (Table 1). The participants in this study provided experiences from across different rehabilitation settings for an array of underlying health concerns. The healthcare professionals varied in their level of experience, but all had at least 5 years of experience working with people who access rehabilitation, and most had 10–20 years of experience and had worked across private and public health settings. Characteristics of the study cohort including the numbers of each profession represented, the health care sector, roles held and clinical indications treated are summarised in Table 1.

**Table 1:** Healthcare professional participant discipline or relevant role.

Discipline or relevant role of participants	*N*	Sector/Role/Clinical indications
Rehabilitation physician	3	Public, design rehabilitation programs, referral to other services, inpatient, aged‐care, stroke, neurological interventions
Orthopaedic surgeon	4	Private, refer to service providers
Neurosurgeon	1	Mostly public, some private, evaluate rehabilitation needs with multidisciplinary team Public and private, Specialist, patient review
General practitioner	1	Private, Chronic disease management, referral to other services
Psychiatrist	1	Public, inpatient acute care
Medical oncologist	1	Public, referral to public and private services
Neurologist	1	Public and private, referral to other services, stroke, migraine
Physiotherapist	8	Public and private, hospital inpatients and community programs, traumatic brain injury, stroke, neuro‐rehab, cardio‐pulmonary
Speech Pathologist	1	Public, community stroke team and aged care, hospital to home transitioning
Dietitian	1	Private, community‐based, diabetes, stroke
Exercise Physiologist	3	Public and private, design novel interventions (research), design rehabilitation programs
Rehabilitation co‐ordinator	1	Public, mixed caseload, inpatient and outpatient care coordinator
Rehabilitation service manager	2	Public, mixed caseload, internal referrals
Multicultural health liaison officer	1	Public, mixed caseload, internal referrals
Clinical telehealth manager	1	Public, support telehealth service provision
Interpreter service manager	1	Public, mixed caseload, interpreting service for rehab patients
Rehabilitation research fellow (scientist)	1	Public, design of novel interventions (research)
Total	31	

### 3.2 Data Analysis

The investigative team considered that the reported prior experiences provided by the designated interviewers (N.H. and G.M.), as rehabilitation professionals or as previous rehabilitation patients, were unlikely to impact their capacity to undertake the face‐to‐face interviews with study participants.

Interview transcripts and preliminary themes identified by the investigative team were sent back to all participants for contextualisation input. The feedback provided from this review indicated that the transcripts generated were an accurate representation of the interviews conducted. In addition, no changes to the themes identified by the researchers were suggested.

#### 3.2.1 Factors Guiding Decisions

Participants reflected on their decision‐making processes in the context of their individual service and the system, including considering guidelines, eligibility, criteria to gain access to programs and the availability of programs. When designing rehabilitation programs, healthcare professionals pointed to using a ‘goal‐focused’ approach, balancing their patient's clinical needs with their personal goals for recovery. Healthcare professionals also reported considering their patient's clinical and social needs, including the logistics required for the patient to attend rehabilitation, the availability of carer support and their employment situation. The importance of combining expertise and advice from multidisciplinary healthcare professionals was recognised and, where available, highly valued. Limitations around accessing multidisciplinary advice, particularly in home or community settings, were acknowledged. Involving the patient and their family or a support person in goal setting to understand the rehabilitation program was reported as beneficial for both recovery and managing expectations along the recovery pathway. For quotations relating to this topic see Table 2, quotes 1, 2, 3.

#### 3.2.2 Keeping Abreast of Information

Two main areas of information to keep abreast were identified: latest clinical evidence and available services for patients. Participants acknowledged that it could be difficult to keep up with the latest clinical evidence and what rehabilitation services were available, citing lack of time and practice changes as key barriers to finding this information. Nonetheless, staying up to date was perceived as critical to ensuring the delivery of high‐quality care, with individual clinics creating links to universities or assigning responsibility to a team member to gather and report on the latest clinical evidence.

Participants cited three main methods that were used to keep up to date: professional networks, digital media and feedback from patients. The professional networks used by healthcare professionals include formal clinical associations that disseminate new developments and provide resources to informal networks of colleagues with greater experiential knowledge. Some healthcare professionals reported that their service had created their own resources for making this information accessible (e.g., lists of local rehabilitation services and programs); however, the maintenance of these resources varied depending on staff and resourcing. Collegial local networks that could be used for advice on relevant and available local programs were highly valued. Beyond using a general internet search, special interest Facebook pages and Twitter accounts were the primary social media platforms cited by healthcare professionals to find relevant information or local rehabilitation programs. Feedback from patients about the different rehabilitation programs, and how helpful they were, was another key method for increasing awareness of local rehabilitation programs. For quotations relating to this topic see Table 2, quotes 4, 5.

#### 3.2.3 Perceived Barriers and Enablers for Gaining Access to Rehabilitation Services

Participants reported that it is difficult to get rehabilitation right when accurate and comprehensive information about both the consumer and the available services is hard to find and at the time when it is needed. They acknowledged that they were often time‐poor and cognitively overloaded by the demands of their role, which impacted their ability to address or overcome these issues in a comprehensive or meaningful way. Healthcare professionals shared their main pain points and what they saw as the greatest areas for improvement: the definition of rehabilitation, access to information for services and patient health data and personalised services.

**Table 2:** Selected quotations relating to each discussion topic or theme identified.

Topic/Theme	Quote number	Quotation	Profession of cited quotation
**Factors guiding decisions**	1	We just follow state‐wide guidelines on who should be accepted in a [subacute ambulatory care service] program.	Rehabilitation Coordinator
	2	[The decision is] sometimes evidence‐based, but then they really also can be really practical, like as in do you work full time?	Physiotherapist
	3	It's really useful for [patients] to have multidisciplinary care. It's easy when there is a structure but not that easy when you are outside of an organised structure.	Neurologist
**Keeping abreast of information**	4	It's such a disservice if you're not aware of what the research evidence is and what the options are – then, you know, patients missed out.	Physiotherapist
	5	I think clients themselves have the best information. They are the ones that do a lot of the legwork in finding those services and the supports.	Speech Pathologist
**Perceived barriers and enablers for gaining access to rehabilitation services**			
**1. Defining ‘Rehabilitation’**	6	It's the question I get the most, ‘What do we need to do post‐surgery? You know do I go home, do I have rehab at home, do I go into inpatient rehab, do I do outpatient rehab?’ There is a lot of confusion about what each of these options mean.	Surgeon
	7	There is a lack of understanding of benefits of plain exercise and/or the common‐denominator parts of what rehab entails…people could just do it themselves.	Medical oncologist
	8	I don't think you can underestimate word of mouth, of that influencing people's opinions. They'd rather go to see [X clinician] if Gladys next door said actually, ‘Oh she's really nice!’ Like they will make choices based on that kind of stuff.	Physiotherapist
**2. Information on access to services and patient health data**	9	…I'm still trying to get a grasp of what's available locally…I mean while websites can help, sometimes they're not localised enough for the patient's needs.	Surgeon
	10	You make that referral, but you don't know that if the person is going to be waiting 3 months to have that referral screened.	Rehabilitation physician
	11	Data about rehab beds and wait times in a digestible format, easily accessible at the right time. Otherwise, you don't know, and it's an awful uncertainty for patients and referring clinicians. There needs to be more predicting and pre‐planning moves to rehab. Work through the temporal disconnect.	Neurosurgeon
	12	Unfortunately, even when you refer them you don't get any feedback, as in how they're going, should we do anything else? That seems to be very dependent on the service, and then you get the patient back afterwards, and then they tell you whether it was beneficial or not.	Rehabilitation physician
**3. The system does not expect patient diversity**	13	Everyone's working Monday to Friday, nine to five, and everyone who needs your services also Monday to Friday nine to five. This is part of the broken system and part of the rehab options that becomes why people don't get the rehab they need.	Exercise physiologist
	14	Lack of flexible or info tailored to people with real‐life problems means people feel like they can't do what's recommended.	Exercise physiologist
	15	… they increase accessibility for people who already had access, and they decrease [access] for people what were already marginalised…often they might get a little more isolated and find it even harder to navigate and find what they need.	Rehabilitation research scientist
	16	… directly asking [patients], I think a lot of it is just left assumed or unsaid and I think actively asking the question “Would they like services, Would they like an interpreter? Would they like a liaison officer?” that goes a long way… what I find is a lot of multicultural groups wouldn't necessarily speak up for themselves.	Surgeon

##### 3.2.3.1 Theme 1: Defining Rehabilitation

Participants reported that the concept of rehabilitation is not well understood by many services, clinicians, patients and the public. This lack of clarity can result in unnecessary, inappropriate and untimely referrals to programs, misunderstanding of the benefits of rehabilitation participation, a desire to partake in programs that may not be suitable and a lack of clarity about whether rehabilitation is relevant to their individual needs. With a clearer definition of what constitutes rehabilitation, healthcare professionals suggested that much to improve recovery can be accomplished at home with the right advice.

Healthcare professionals reported that that their patients were typically enthusiastic about taking part in rehabilitation, but that they often do not understand whether available options are suitable for their specific needs. They also reported that some patients had strong views and preferences that were not necessarily evidence‐based, which could lead to ineffective programs and services. For quotations relating to this theme, see Table 2, quotes 6, 7, 8.

##### 3.2.3.2 Theme 2: Information on Access to Services and Patient Health Data

Regardless of speciality, participants cited the importance of timely access to services that offered the most appropriate rehabilitation needs for each patient. This, however, requires knowledge and understanding of the available local services, which some participants reported as unavailable or difficult to source, particularly when new to an organisation or locality or services were in more than one site. This was generally overcome through word of mouth within their unit/department (e.g., from colleagues) or where their own services had established their own resources. Even when healthcare professionals were aware services existed, they identified gaps in their ability to easily access information about the availability of these programs, for example, bed vacancies and wait times.

Participants reported a desire to have better communication pathways between their own services and those providing rehabilitation to have complete information on the patient and other relevant medical information, for example, their medical history and comorbidities. They recognised that more information would also facilitate better monitoring of patient progress and inform ongoing care decisions. In the absence of or without easy access to patient health information, healthcare professionals felt unable to ensure the provision of personalised, high‐value, goal‐focussed rehabilitation programs. Without access to individual patient data, healthcare professionals felt unable to evaluate the service's effectiveness, which could inform referrals for future patients with similar needs. Instead, they often relied on direct objective feedback from the patient to gather this information. For quotations relating to his theme see Table 2, quotes 9, 10, 11, 12.

##### 3.2.3.3 Theme 3: The System Doesn't Expect Patient Diversity

Healthcare professionals recognised the diversity of their patients' clinical and personal needs and goals for their rehabilitation program and acknowledged that processes, systems and services were often not catered for in the ‘standard package of services’ provided routinely. Beyond the presenting clinical situation, this diversity extended to the availability to participate in set rehabilitation programs, competing responsibilities, psycho‐social needs, financial capacity, cultural needs, health literacy, digital literacy, access to digital technologies, language barriers and physical transportation. These needs can also vary greatly over time for a patient, meaning some patients may miss out on accessing the care they need. Limited available service options and supports (including financial programs) tailored to individual needs were shown to be a barrier to patients receiving appropriate care. With the high demand for medical rehabilitation services, healthcare professionals felt those with more ‘straightforward’ needs typically had more options for services, easier access to services and therefore more often received timely and appropriate care. For quotations relating to this theme, see Table 2, quotes 13, 14, 15, 16.

## 4 Discussion

In the *Rehabilitation Choices* study, we explored with rehabilitation healthcare professionals the current landscape of decision‐making, enablers and barriers to accessing appropriate rehabilitation services in the Australian setting. We explored factors that influence healthcare professionals' decisions around their and their patients' engagement with medical rehabilitation services and sought to identify elements that facilitate service provision or were perceived to be barriers to the provision of appropriate services for their patients. This exploratory approach with a multidisciplinary group of healthcare professionals working across varied healthcare settings enabled us to identify those factors common to different health conditions and healthcare settings. This approach recognises that rehabilitation typically does not occur as a siloed event, with patients moving across different services and over an extended timeframe as part of their rehabilitation journey. Identifying and embracing those enablers and addressing barriers commonly experienced by all rehabilitation healthcare professionals provides a target where broad‐scope improvements can be made. This will support healthcare specialists and services in providing timely, accessible and appropriate rehabilitation to patients to support optimal recovery.

One barrier that was identified by healthcare professionals to providing good services was challenges in keeping up to date with information relating to (i) available and relevant local rehabilitation services and (ii) changes to clinical guidelines/best practices. They recognised both as key facilitators to ensure the delivery of high‐quality care to their patients. This view has been reflected by others, in the setting for stroke rehabilitative services [30, 31] and other chronic diseases [32]. In the current study, healthcare professionals expressed their willingness to engage with new information and resources that could help them both refer to and provide best‐practice rehabilitative care, should these become available. Our recommendation would be the potential creation of a web‐based rehabilitation service guide that maps local services and provides information about programs and clinical expertise available.

A further barrier that was reported by healthcare professionals in the current study was a lack of clarity around the definition of rehabilitation. Participants reported a desire for a clear common definition to improve understanding and communication between services, clinicians and patients to optimise the recovery journey and manage patient expectations. Without a clear definition of rehabilitation, it can be difficult for therapists to explain, and patients to understand, what services are available and appropriate. For the patient, this can become understandably frustrating, upsetting and potentially lead to disengagement from their recovery efforts. Patients' perceptions of poor or inconsistent communication from their healthcare providers can negatively impact confidence, satisfaction, treatment adherence, outcomes and overall wellbeing [33]. Conversely, a good patient–provider relationship and open communication [17] and engagement in realistic goal setting [30] have been shown to motivate the patient and facilitate rehabilitation and recovery.

The lack of a clear definition of rehabilitation has been recognised by the WHO, which recently provided a consensus definition of rehabilitation [1, 2]. The need for a clear definition of rehabilitation has been illustrated in several settings. For some rehabilitation specialities, such as in pulmonary medicine rehabilitation, the concept of disease‐specific rehabilitation is not widely recognised, highlighted in a study undertaken looking at factors that influence the referral practices of Australian general practitioners to rehabilitation services for COPD patients [13]. There was a lack of knowledge regarding not only the type of services that constitute ‘rehabilitation’ that can be accessed but also the expected outcomes that could be achieved. In a subsequent review regarding pulmonary medicine rehabilitation [34], the most common barriers to rehabilitation were again seen as a lack of knowledge of what a pulmonary medicine rehabilitation service would comprise, how to refer patients and outcomes that could be achieved.

A lack of clarity around the definition of rehabilitation may also help in ensuring that therapists and services are optimally utilised. For example, in an international study exploring referrals to physiotherapists, there was an underutilisation of services in low‐ and middle‐income countries, which in part was attributed to a lack of recognition of the therapists' role in rehabilitation [35]. The ambiguity surrounding the scope, role and achievable outcomes of attendance at rehabilitation services has also been reported in a recent Australian study, in which healthcare professionals noted challenges in service provision were largely due to unclear role definitions, a lack of interdisciplinary teamwork and a lack of clarity of rehabilitation outcomes [25].

In the current study, rehabilitation health professionals reported a desire for access to individual patient data from multiple providers to make informed decisions regarding appropriate rehabilitation service referrals, and also to monitor patient progress to inform ongoing care. The desire for better access to individual patient data is not specific to the rehab setting, with better data access a priority of the national and state health departments across Australia. A similar finding has been reported from Canada, with rehabilitation specialists and health care providers proposing access to centralised patient information, and better links between facilities and providers to improve patient management [17]. National efforts are underway to improve patient health data access across Australia; however, efforts are hampered by confidentiality, data access/linking, data cleaning and cyber security challenges.

A lack of information on the availability and quality of services means that many clinicians are compelled to make choices that may not be the best in terms of meeting their patient's needs, preferences and expectations. Indeed, findings from a systematic review of barriers to rehabilitation following total joint replacement highlight the influence of a complex interplay between clinical, social and psychological features of patients on their engagement in rehabilitation programs [36]. This highlights the need for a model of patient‐centric care in which these patient‐specific features of services are communicated to health professionals to support informed, bidirectional communication between providers and patients. This information can facilitate matching patients to services that can appropriately support their needs.

The needs of the patient in determining service suitability are increasingly being highlighted. For example, in their 2022 report, the Australian Stroke Foundation stated that rehabilitation delivery should be a proactive, person‐centred and goal‐oriented process for consumers [11]. Moreover, the Australian Royal College of Surgeons has suggested that patients and carers should be informed and encouraged to access rehabilitation services, but structural changes to the care pathway and incentives are needed, to support surgeons in providing more information to patients and promoting rehabilitation in appropriate settings [36]. When personalised support and greater awareness about rehabilitation are provided, improved physical and psychosocial outcomes have been observed [28]. The findings from the current study suggest a desire for health professionals to better match their patients with appropriate services, in line with recommendations; however, processes and infrastructure to support across different service settings are missing. The creation of such resources would be to consider the needs of patients with complex rehabilitation needs, such as those recovering from stroke, where rehabilitation requires a multidisciplinary team and the recovery pathway is prolonged [31, 37] and transitions from the hospital to the home environment [38]. New systems and processes, some currently being developed, which integrate single patient data records with details of available hospital and community resources will be essential to address this challenge.

### 4.1 Strengths and Limitations

In the current study, we looked at a broad cross‐section of healthcare professionals involved with the provision of, or referral to, rehabilitation services. In doing so, we identified general themes that were expressed the most by professionals across a wide variety of roles and clinical indications. We see this as a strength of the study, as the identification of barriers and enablers to rehabilitation service provision across this broad cross‐section of health professionals can inform overarching policies, with the potential to have the widest impact. However, in doing so, our findings may limit details of more nuanced factors encountered in specific professions or apply to specific clinical groups. Despite this, we did observe that many of the themes that emerged from our interviews were mirrored by those achieved in studies derived from healthcare professionals working in specific clinical indications such as stroke [30], COPD [39], joint replacement [12] and oncology [40] undertaken by others, giving our findings validity when considering strategies to support facilitators and minimise the barriers, impacting on rehabilitation service provision from the healthcare professionals perspective.

## 5 Conclusions

This study aimed to understand how healthcare professionals make decisions about referring to and offering rehabilitation, what they perceive makes it easy or hard for patients to access the rehabilitation they need and what they think could make things better. Many of the themes reported in the current study mirror those seen in studies conducted in other countries despite differences in referral processes or healthcare systems.

This paper, Part 1 of 2, provides a summary of the identified needs of healthcare providers of medical rehabilitation services in the *Rehabilitation Choices* study. Several key barriers that impact rehabilitation service referral and provision by a broad cross‐section of healthcare professionals were identified including issues regarding the definition of rehabilitation and the need to have access to up‐to‐date information, improved communication with patients and rehabilitation providers and needing an infrastructure that provides the capacity for personalized service provision.

## Author Contributions

**Nicolette Hodyl**: investigation, writing–original draft, methodology, writing–review and editing, project administration, formal analysis, data curation, supervision. **Gillian Mason**: investigation, writing–original draft, methodology, writing–review and editing, formal analysis, project administration. **Karen Ribbons**: writing–original draft, writing–review and editing. **Lucy Bailey**: formal analysis, project administration. **Adrian O'Malley**: investigation, writing–review and editing, formal analysis. **Tracy Ward**: investigation, writing–review and editing, formal analysis. **Stephen Ward**: investigation, writing–review and editing, formal analysis. **Michael Pollack**: conceptualization, writing–review and editing, supervision. **Michael Nilsson**: conceptualization, funding acquisition, writing–review and editing, supervision. **Frederick Rohan Walker**: supervision, conceptualization, funding acquisition, writing–review and editing.

## Conflicts of Interest

The authors declare no conflicts of interest.

## Data Availability

The data that support the findings of this study are available on request from the corresponding author. The data are not publicly available due to privacy or ethical restrictions.
